# Controlling *Streptococcus mutans* and *Staphylococcus aureus* biofilms with direct current and chlorhexidine

**DOI:** 10.1186/s13568-017-0505-z

**Published:** 2017-11-15

**Authors:** Hao Wang, Dacheng Ren

**Affiliations:** 1 Department of Biomedical and Chemical Engineering, Syracuse University, Syracuse, NY 13244 USA; 2 Syracuse Biomaterials Institute, Syracuse University, Syracuse, NY 13244 USA; 3 Department of Civil and Environmental Engineering, Syracuse University, Syracuse, NY 13244 USA; 4 Department of Biology, Syracuse University, Syracuse, NY 13244 USA

**Keywords:** Biofilm, Electrochemical control, Chlorhexidine, Synergistic effects

## Abstract

Microbial biofilms formed on biomaterials are major causes of chronic infections. Among them, Gram-positive bacteria *Streptococcus mutans* and *Staphylococcus aureus* are important pathogens causing infections associated with dental caries (tooth-decay) and other medical implants. Unfortunately, current antimicrobial approaches are ineffective in disrupting established biofilms and new methods are needed to improve the efficacy. In this study, we report that the biofilm cells of *S. mutans* and *S. aureus* can be effectively killed by low-level direct current (DC) and through synergy in concurrent treatment with DC and chlorhexidine (CHX) at low concentrations. For example, after treatment with 28 µA/cm^2^ DC and 50 µg/mL CHX for 1 h, the viability of biofilm cells was reduced by approximately 4 and 5 logs for *S. mutans* and *S. aureus,* respectively. These results are useful for developing more effective approaches to control pathogenic biofilms.

**Electronic supplementary material:**

The online version of this article (10.1186/s13568-017-0505-z) contains supplementary material, which is available to authorized users.

## Introduction

Biofilms are formed by microbial cells embedded in a matrix comprised of extracellular polymeric substance (EPS) containing polysaccharide, proteins, and DNA. The presence of this extracellular matrix provides protection to microbial pathogens from antimicrobials to host immune cells/factors (Liu et al. 2016; Hall and Mah 2017). Biofilms can form on both biotic and abiotic surfaces and are common causes of chronic infections including dental plaques (Smith et al. 2011; Song et al. 2015). The protection of EPS plus the dormancy of biofilm cells render these multicellular structures extremely difficult to eradicate (Kouidhi et al. 2015; Smith et al. 2011; Song et al. 2015).

*Streptococcus mutans* is a Gram-positive bacterium commonly found in human dental biofilms. It is a dominant species with higher biomass in dental biofilms than other *Streptococcus* species, including *S. sanguinis, S. mitis*, and *S. salivarius*, due to its acid tolerance and thus the capability to live in low pH environment of oral cavities (Bender et al. 1986; Harper and Loesche 1984; Kreth et al. 2005). *S. mutans* expresses multiple exoenzymes (glucosyltransferases) that make it the primary EPS producer in oral cavity (Falsetta et al. 2014), while it is also highly acidogenic and aciduric. *S. mutans* can rapidly colonize tooth surface and establish cariogenic biofilms with extracellular polysaccharides (EPS). This acidifies the local microenvironment and promotes the growth of an acidogenic microbiota, facilitating the development of dental caries (Falsetta et al. 2012, 2014).

*Staphylococcus aureus* is also an abundant Gram-positive bacterium, which usually harbors in the nasal passages and ears of patients (Smith et al. 2011). Previous studies have shown that *S. aureus* is not only a significant cause of many localized and systemic infections such as osteomyelitis (Lew and Waldvogel 2004), chronic wound infection (Hansson et al. 1995), and chronic rhinosinusitis (Stephenson et al. 2010), but also has a strong connection to dental implant infections (Salvi et al. 2008; Harris et al. 2004). The established biofilms of *S. aureus*, especially the methicillin-resistant *S. aureus* (MRSA), are highly tolerant to common antimicrobial treatments (Jones et al. 2001; O’Donnell et al. 2015; Lewis et al. 2015).

Few approaches are currently available for controlling cariogenic biofilms (Liu et al. 2016). Chlorhexidine (CHX) is considered the “gold standard” for oral antimicrobial therapy (Jones 1997). However, use of high dose CHX has adverse side effects such as tooth staining and calculus formation. Also, CHX is not recommended for long term daily therapeutic use (Flotra et al. 1971). In 1994, Costerton et al. (1994) reported bacterial killing by synergistic effects between low-level electric currents and antibiotics, a phenomenon named “bioelectric effects”. Since 1990s, direct currents (DCs) ranging from μA to mA have been reported for their bactericidal effects after a relatively long period (from several hours to days) of treatment time (Costerton et al. 1994; del Pozo et al. 2009; Schmidt-Malan et al. 2015; Spadaro et al. 1974) either by DC alone or with antibiotics together (Wattanakaroon et al. 2000; Niepa et al. 2012, 2016a). Recent studies reported that mA level DC could enhance the killing effect of 0.2% (200 µg/mL) chlorhexidine on biofilms of Gram-negative *Porphyromonas gingivalis*, although there was no bactericidal effect by DC alone (Lasserre et al. 2015). To explore the potential of lower levels of DC and CHX in killing dental biofilms of Gram-positive bacteria, we conducted this study using *S. mutans* and *S. aureus* as model species. We demonstrate that stainless steel electrode derived DC and CHX have strong synergy in killing *S. mutans* and *S. aureus* biofilms; and the levels of DC and CHX appear to be lower than other reported systems.

## Materials and methods

### Bacteria strains and growth media

*Staphylococcus mutans* Clarke strain (ATCC 25175) was cultured in brain heart infusion (BHI) broth (BD Biosciences, San Jose, CA, U.S.) (Murchison et al. 1982). The *S. aureus* ALC2085 (strain RN6390 containing pALC2084) was obtained from Dr. Karin Sauer at Binghamton University (Sauer et al. 2009) and cultured in Lysogeny broth (LB) (Sambrook and Russell 2001) containing 10 g/L tryptone, 5 g/L yeast extract, and 10 g/L NaCl, supplemented with 10 µg/mL chloramphenicol (Sigma-Aldrich, St. Louis, MO, U.S.). Both strains were routinely cultured overnight at 37 °C with shaking at 200 rpm.

### Biofilm formation

Biofilms were formed on acrylic coupons (3.5 cm × 0.5 cm × 0.1 cm; McMaster-Carr, Aurora, OH, U.S.). Briefly, 25 µL of an overnight culture of *S. mutans* was used to inoculate a petri dish containing 25 mL of BHI medium and acrylic coupons. The culture was incubated at 37 °C for 48 h without shaking. Then the coupons with biofilms were removed from petri dish and washed three times with 0.85% NaCl solution to remove all planktonic cells and only retained the firmly attached cells for DC and CHX treatments. The *S. aureus* biofilm samples were prepared in the same way except that the medium was LB plus 10 µg/mL chloramphenicol and the incubation time was reduced to 24 h due to a higher growth rate of *S. aureus*.

### Electrochemical treatment

The experimental system for DC treatment is the same as we described previously (Niepa et al. 2012, 2016b). Briefly, an electrochemical cell was constructed with two electrodes on the opposite sides of a plastic cuvette (Thermo Fisher Scientific, Pittsburg, PA, U.S.). DC was generated using a potentiostat (Potentiostat WaveNow, Pine Research Instrumentation, Raleigh, NC, U.S.) in the three electrode system with a silver wire (0.015” diameter, A-M Systems, Sequim, WA, U.S.) placed in bleach for 30 min to create an Ag/AgCl reference electrode. The DC level and voltage across the electric field were monitored and recorded using the AfterMath software (Potentiostat WaveNow, Pine Research Instrumentation, Raleigh, NC, U.S.) in the galvanostatic mode during the treatment.

### DC treatment of biofilms

Each DC treatment was carried out in 3 mL 0.85% NaCl solution. First, a sterile SS304 electrode (3.5 cm × 0.95 cm × 0.05 cm) was inserted into a cuvette, followed by an acrylic coupon with *S. mutans* or *S. aureus* biofilm attached. Another sterile SS304 electrode was then inserted on the opposite side. The biofilm was treated galvanostatically with direct current (DC) for 1 h in the absence or presence of CHX (MP Biomedicals, Solon, OH, U.S.). Samples treated with DC or CHX alone and untreated samples were used as controls. After treatment, each acrylic coupon was transferred to a 10 mL tube containing 5 mL 0.85% NaCl solution. The biofilm cells were removed from the surface by gentle sonication for 1 min. The number of viable cells detached from acrylic coupons was quantified by counting colony forming units (CFUs) in the solution.

To further evaluate the effects in an environment similar to that of oral cavity, the test medium was replaced with artificial saliva medium or a mixture of 0.85% NaCl and artificial saliva medium (2:1). The recipe of artificial saliva from Pratten et al. (1998) was followed. It contains 2 g/L yeast extract, 5 g/L peptone, 2.5 g/L type III hog gastric mucin, 0.2 g/L NaCl, 0.2 g/L KCl, and 0.3 g/L CaCl_2_, supplemental with 1.25 mL of sterile 40% urea. The CHX was tested at 50 µg/mL to 500 µg/mL. The treatment process was the same as described above for 0.85% NaCl solution.

### Live/dead staining

To corroborate the CFU results, another set of acrylic coupons with biofilms treated with DC and CHX in the same way were stained with Live/Dead staining kit (Life Technologies Inc., Carlsbad, CA, U.S.) for 10 min. Then the biofilm samples were imaged using a fluorescence microscope (Axio Imager M1, Carl Zeiss Inc., Berlin, Germany).

### Statistical analysis

All data are presented as mean ± standard deviation. Statistical significance was assessed with two-way ANOVA followed by Tukey test. Statistical significance was set as *p* < 0.05. All analyses were performed using SAS 9.4 software (SAS Institute, Cary, NC, USA).

## Results

### Effects of DC and CHX on *S. mutans* and *S. aureus* biofilms in 0.85% NaCl solution

**Fig. 1:**
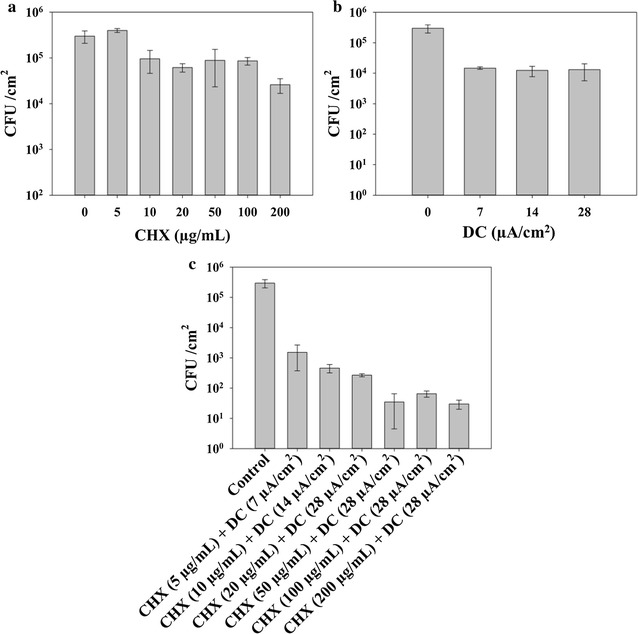
Viability of *S. mutans* biofilm cells after 1 h treatment. **a** Treatment with CHX alone. **b** Treatment with DC alone. **c** Concurrent treatment with CHX and DC. All treatments were tested in 0.85% NaCl solution

**Fig. 2:**
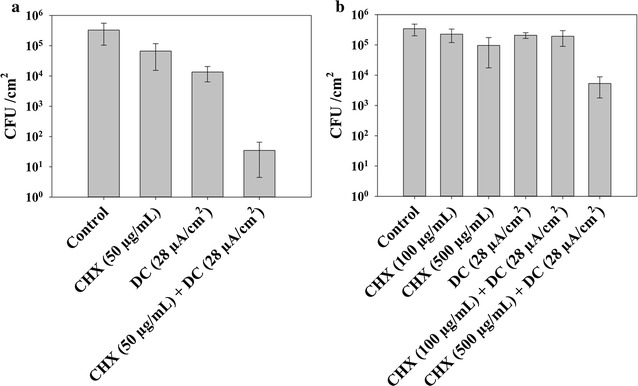
Viability of *S. mutans* biofilm cells after treatment with CHX, DC, or concurrent treatment. **a** Treatment in 0.85% NaCl. DC level: 28 µA/cm^2.^ CHX dosage: 50 µg/mL. **b** Treatment in artificial saliva. DC level: 28 µA/cm^2^. CHX dosage: 100 or 500 µg/mL

As shown in Fig. 1, treatment with either CHX (at 5, 10, 20, 50, 100 and 200 µg/mL, Fig. 1a) or DC (at 7, 14 and 28 µA/cm^2^, Fig. 1b) showed moderate but statistically significant killing (*p* < 0.05, two-way ANOVA followed by Tukey test). For example, up to 1.2 log and 0.7 log of killing was obtained with 28 µA/cm^2^ DC and 50 µg/mL CHX, respectively. Furthermore, synergy was observed between DC and CHX in killing *S. mutans* biofilms dose dependently. Among the tested conditions, the maximum killing effect (4 logs) was observed under the condition of 28 µA/cm^2^ DC and 50 µg/mL CHX (*p* = 0.02, two-way ANOVA followed by Tukey test; Figs. 1c, 2a). The higher concentration of CHX (100 and 200 µg/mL) did not show significant increase in killing of *S. mutans* biofilm cells (compared to 50 µg/mL) both in the absence and presence of DC (*p* > 0.6, two-way ANOVA followed by Tukey test; Fig. 1a, c).

**Fig. 3:**
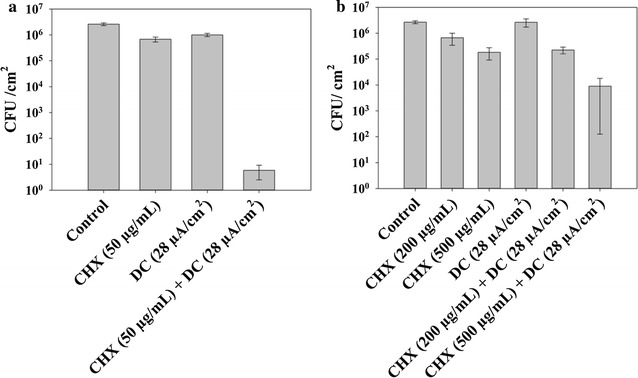
Viability of *S. aureus* biofilm cells after treatment with CHX, DC, or concurrent treatment. **a** Treatment in 0.85% NaCl. DC level: 28 µA/cm^2^. CHX dosage: 50 µg/mL. **b** Treatment in artificial saliva. DC level: 28 µA/cm^2^. CHX dosage: 200 or 500 µg/mL

Similar synergistic effects were also observed for *S. aureus* biofilms under the same treatment conditions. The number of viable *S. aureus* biofilm cells was reduced by more than 5 logs (*p* < 0.0001, two-way ANOVA with Tukey test; Fig. 3a) after treatment with 28 µA/cm^2^ DC and 50 µg/mL CHX for 1 h in 0.85% NaCl solution. In comparison, treatment with the same level of DC or CHX alone only reduced the number of viable biofilm cells by 60.0 ± 7.9 and 74.3 ± 2.5% (less than 1 log for both conditions), respectively.

**Fig. 4:**
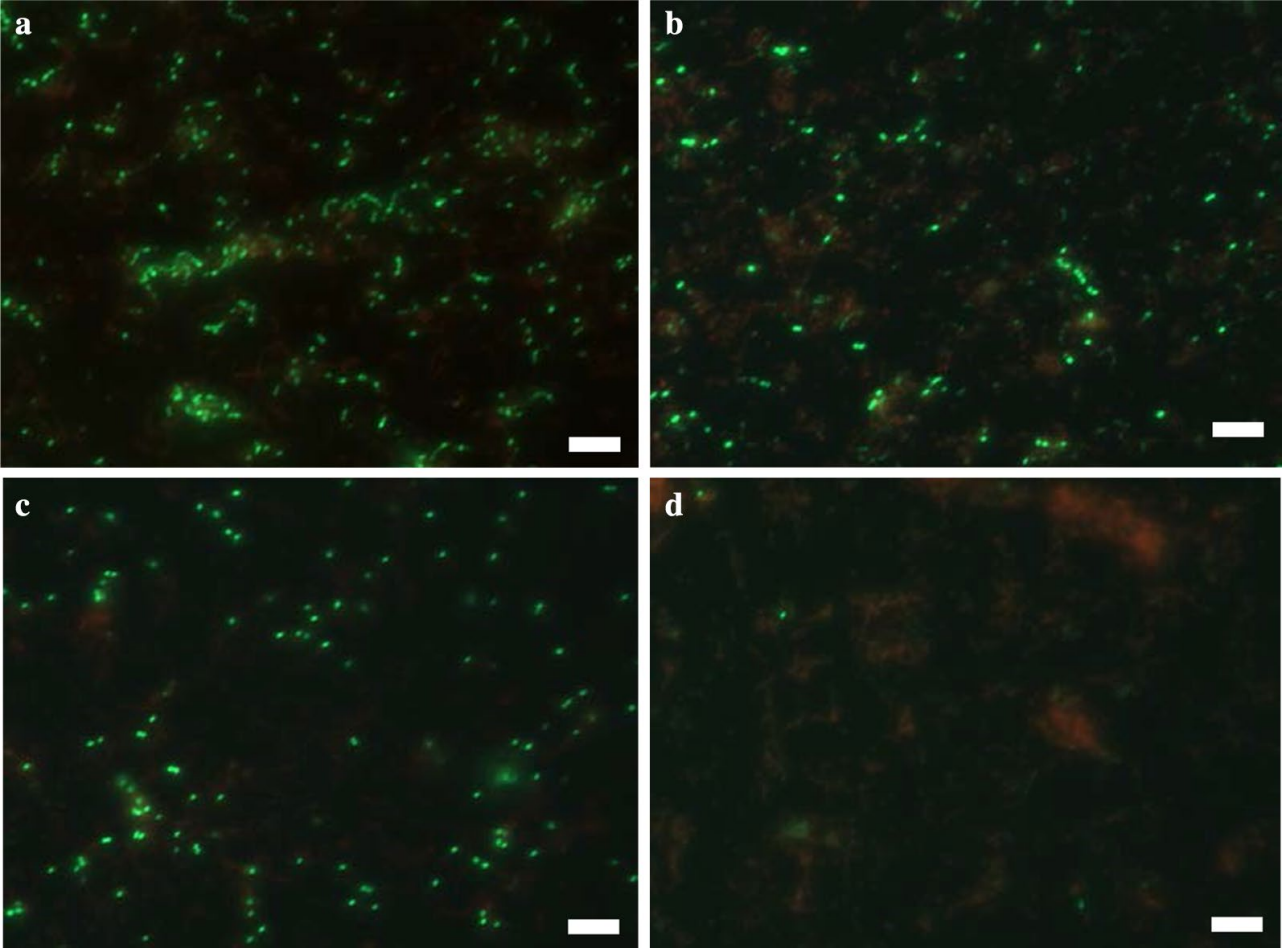
Live/dead staining of *S. mutans* biofilms after treatment with CHX, DC, or concurrent treatment. **a** Without treatment. **b** Treatment with 5 μg/mL CHX. **c** Treatment with 7 μA/cm^2^ DC. **d** Concurrent treatment with 5 μg/mL CHX plus 7 μA/cm^2^ DC. Bar = 20 μm

**Fig. 5:**
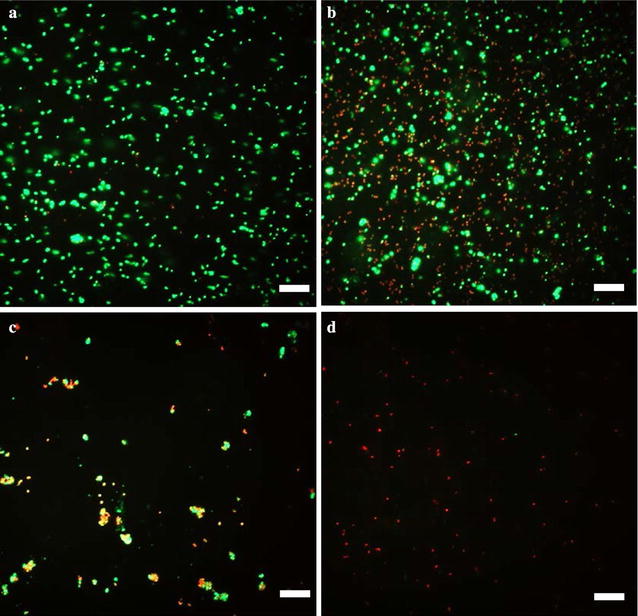
Live/dead staining of *S. aureus* biofilms after treatment with CHX, DC, or concurrent treatment. **a** Without treatment. **b** Treatment with 20 μg/mL CHX. **c** Treatment with 28 μA/cm^2^ DC. **d** Concurrent treatment with 20 μg/mL CHX and 28 μA/cm^2^ DC. Bar = 20 μm

The CFU results were corroborated with fluorescence microscopy. According to the images from Live/Dead staining of *S. mutans* and *S. aureus* biofilms, the number of live cells (green) decreased when treated with DC and CHX even at low doses (7 µA/cm^2^ DC and 5 µg/mL CHX for *S. mutans*, 28 µA/cm^2^ DC and 20 µg/mL CHX for *S. aureus*); and almost no live cells (green fluorescence only) were found on the surface of acrylic coupons after concurrent treatment with DC and CHX together (Figs. 4, 5). Compared with sample treated with DC alone, samples treated with both CHX and DC concurrently only have patches of cell debris in red, suggesting that substantial cell lysis might have occurred.

During treatment, we also observed that some small particles and tiny gas bubbles were released from anode to cathode, respectively. The particles are metal oxides as we reported in our previous study on *P. aeruginosa* under the same experimental setup (Niepa et al. 2012, 2016a). Based on the half reaction potential of related species (Lide 2006), we speculate that hydrogen peroxide was generated by the reduction reaction of the oxygen in the solution. It will be interesting to further characterize these reactions and link the electrochemical reactions products to the killing effects. This is part of our ongoing work.

### Effects in the presence of artificial saliva

Since the surface of dental implants is commonly covered with saliva, we also tested the effects of DC and CHX in the presence of artificial saliva. When artificial saliva was added to 0.85% NaCl solution as treatment medium, the killing effects were reduced but still significant (*p* < 0.05, two-way ANOVA followed by Tukey test; Additional file 1: Figures S1 and S2). For example, the reduction of biofilm cell viability was 98.0 ± 0.4% (~ 1.7 log) when *S. aureus* biofilm was treated with 50 µg/ml CHX and 28 µA/cm^2^ DC in a mixture of artificial saliva and 0.85% NaCl solution (1:2 v/v) (Additional file 1: Figure S1). No killing effect was observed when biofilms were treated in pure artificial saliva medium under the same dosage of CHX or DC level. However, when the concentration of CHX increased to 500 µg/mL (0.05 w/v%; the dosage used in commercial oral rising products is 0.12 w/v%) while keeping the DC level at 28 µA/cm^2^, the number of viable *S. aureus* cells in biofilm was reduced by 2.5 logs compared to untreated control (*p* = 0.005, two-way ANOVA followed by Tukey test; Fig. 3b). The viability of biofilm cells treated with CHX alone was reduced by approximately 1 log and no significant killing effect was observed for 28 µA/cm^2^ DC treatment alone (Fig. 3b). Similar results were observed for *S. mutans* biofilms (Fig. 2b and Additional file 1: Figure S1), although the killing of *S. mutans* biofilm cells in artificial saliva medium was lower than *S. aureus*. The number of viable cells was reduced by 0.54 log, 0.17 log, and 1.63 log when treated with CHX alone, DC alone, or concurrent treatment with CHX and DC, receptively (*p* = 0.02, two-way ANOVA followed by Tukey test; Fig. 2b).

## Discussion

Direct currents and alternative currents (AC) are known to kill biofilm cells in the presence or absence of antibiotics, and treatment time tested to date varies from hours to days (del Pozo et al. 2009; Schmidt-Malan et al. 2015; Spadaro et al. 1974). Our group recently found synergetic effect between low level DC and the antibiotic tobramycin in killing *Pseudomonas aeruginosa* biofilm and persister cells (Niepa et al. 2012; 2016b). However, most of previous studies focus on biofilms formed directly on the surface of electrodes.

To mimic real applications, it is important to test biofilms that are not in direct contact with electrodes. In this study, we set a sandwich structure with biofilms formed on acrylic coupons in the middle of the electric field and about 1.5 mm from each electrode. Our results show that the viability of *S. mutans* and *S. aureus* biofilm cells (placed between two electrodes) on the surface of denture material can be reduced by low level DC and CHX through concurrent treatment in 1 h; and the effect was approximately 1–3 logs stronger than that obtained with the same level of DC or CHX alone indicating synergistic effects between DC and CHX in killing biofilm cells of these two dental bacteria. The effect was more profound in 0.85% NaCl solution than in the artificial saliva medium. The images of Live/Dead staining also confirmed that there was profound killing by concurrent treatment. We speculated that mucin and other proteins in artificial saliva medium might repress the killing effects of CHX and DC, since DC kills cells partially through the generation and movement of reactive species from electrochemical reactions (Niepa et al. 2012).

We speculated that this synergy was primarily resulted from the interaction between the products of DC treatment and CHX. In the recent studies, it showed that hydrogen peroxide was generated from electrode surface during electrical treatment of bacteria biofilms (Istanbullu et al. 2012; Sultana et al. 2015), which had been reported for its synergetic antibacterial effect with CHX against *streptococcus* and *staphylococcus* species (Steinberg et al. 1999). Furthermore, some metal ions (Zn^2+^, Cu^2+^) were shown for their capabilities to enhance the effect of CHX on different oral pathogens (Cronan et al. 2006; Drake et al. 1993). The stainless-steel electrodes used in this study have a larger surface area and can release multiple types of metal ions including Fe^2+^, Fe^3+^, Cr^2+^, Cr^3+^ and Cr^6+^ during DC treatment (Niepa et al. 2012). Fe^2+^ and Fe^3+^ ions were found to kill *P. aeruginosa* persister cells in the presence of antibiotics in an electric field (Niepa et al. 2016a). Our lab also found that Cr^3+^ and Cr^6+^ can form complex with certain antibiotic compounds, and thus increase the affinity between antibiotics and intracellular targets (Niepa et al. 2016b). It is possible that some released ions interact with CHX molecules and result in the observed synergy in killing *S. mutan*s and *S. aureus*. This is part of our ongoing study.

Recently, Lasserre et al. (2015) reported that the viability of *P. gingivalis* biofilm could be reduced by 81.1 and 98.9% in 10 min when treated with 2000 µg/mL (0.2 w/v%) CHX alone and concurrent treatment with same dosage of CHX and 5882 µA/cm^2^ DC, receptively; while the treatment with DC itself did not kill *P. gingivalis* cells. The biofilms were cultured on the discs of a Modified Robbins Device (MRD), which were placed between two electrodes of platinum wires in the MRD’s chamber. This is an exciting discovery, but the DC level appears high and may not be suitable for in vivo therapy, especially for the implants close to nervous systems that do not tolerate more than a maximum current density of 30 µA/cm^2^ (McCreery et al. 1990; Shannon 1992; Clark 2003). Hence, it is necessary to reduce DC to µA level for future in vivo applications. In this study, we treated *S. aureus* and *S. mutans* biofilm without direct contact to electrodes by placing an acrylic coupon in the middle of a low-level electric field and parallel to the electrode surfaces. By using stainless steel as electrode material, the level of DC and CHX in our study are much lower (28 µA/cm^2^ DC and 50 µg/mL CHX), and strong killing effects (3–4 logs) were obtained.

CHX is bacteriostatic at low concentrations by affecting the integrity of bacterial cell wall and bactericidal at high concentrations by disrupting the cell (McDonnell and Russell 1999). *S. mutans* and *S. aureus* appear to be quite susceptible to CHX according to MIC data (< 8 μg/mL) (Chung et al. 2006). However, the maximum killing of preformed biofilms by CHX alone in our experimental system was only less than 1.5 logs even with a dosage up to 500 μg/mL.

Through synergy with DC, CHX was found to be more effective in killing biofilm cells. The effective doses of CHX we used were only 50 μg/mL (0.005 w/v%) in 0.85% NaCl solution and 500 μg/mL (0.05 w/v%) in artificial saliva medium. This CHX level is expected to be safe because the commercial products for oral wash have approximately 1200 µg/mL (0.12 w/v%)–2000 µg/mL (0.2 w/v%) of CHX. The exact mechanism for such synergistic killing is unknown and is part of our ongoing research.

In summary, we demonstrated that the biofilm cells of two Gram-positive pathogenic bacteria, *S. mutans* and *S. aureus*, could be efficiently killed by concurrent treatment with low level DC and CHX in 1 h. This electrochemical control is effective against the biofilms formed on the acrylic materials. The synergistic effect between DC and CHX can help design new devices and strategies for controlling pathogenic biofilms. The interaction between electrochemical products and CHX may play a significant role in the observed synergy in biofilm killing, which deserves further study.

## Additional file

**Additional file 1: Figure S1.** Viability of *S. aureus* biofilm cells after 1 h treatment with 50 μg/mL CHX alone, 28 μA/cm^2^ DC alone or concurrent treatment with CHX and DC. The treatments were tested in a mixture of 0.85% NaCl and artificial saliva medium (2:1, v/v). **Figure S2.** Viability of *S. mutans* biofilm cells after 1 h treatment with 50 μg/mL CHX alone, 28 μA/cm^2^ DC alone or concurrent treatment with CHX and DC. The treatments were tested in a mixture of 0.85% NaCl and artificial saliva medium (2:1, v/v).

## Abbreviations

AC: alternative currents
ANOVA: analysis of variance
BHI: brain heart infusion
CHX: chlorhexidine
DC: direct current
EPS: extracellular polysaccharides
MRD: modified Robbins device
LB: lysogeny broth
*S. aureus*: *Staphylococcus aureus*
*S. mutan*s: *Streptococcus mutans*
