# Supplementary material for: Controlling Streptococcus mutans and Staphylococcus aureus biofilms with direct current and chlorhexidine
Source: AMB Express. 2017 Nov 15;7:204. doi: 10.1186/s13568-017-0505-z (PMC5688048; doi:10.1186/s13568-017-0505-z)
Supplement: Supplementary file 1 — Additional file 1: Figure S1. Viability of S. aureus biofilm cells after 1 h treatment with 50 μg/mL CHX alone, 28 μA/cm2 DC alone or concurrent treatment with CHX and DC. The treatments were tested in a mixture of 0.85% NaCl and artificial saliva medium (2:1, v/v). Figure S2. Viability of S. mutans biofilm cells after 1 h treatment with 50 μg/mL CHX alone, 28 μA/cm2 DC alone or concurrent treatment with CHX and DC. The treatments were tested in a mixture of 0.85% NaCl and artificial saliva medium (2:1, v/v). [file 13568_2017_505_MOESM1_ESM.pdf]

Additional File

for

Controlling *Streptococcus mutans* and *Staphylococcus aureus* Biofilms with Direct Current and Chlorhexidine

Hao Wang[1, 2] and Dacheng Ren[1, 2, 3, 4*]

[1]Department of Biomedical and Chemical Engineering, Syracuse University, Syracuse, NY 13244,

[2]Syracuse Biomaterials Institute, Syracuse University, Syracuse, NY 13244,

[3]Department of Civil and Environmental Engineering, Syracuse University, Syracuse, NY 13244,

[4]Department of Biology, Syracuse University, Syracuse, NY 13244

***Corresponding author:**

Dacheng Ren: Phone +1-315-443-4409. Fax +1-315-443-9175. Email : dren@syr.edu.

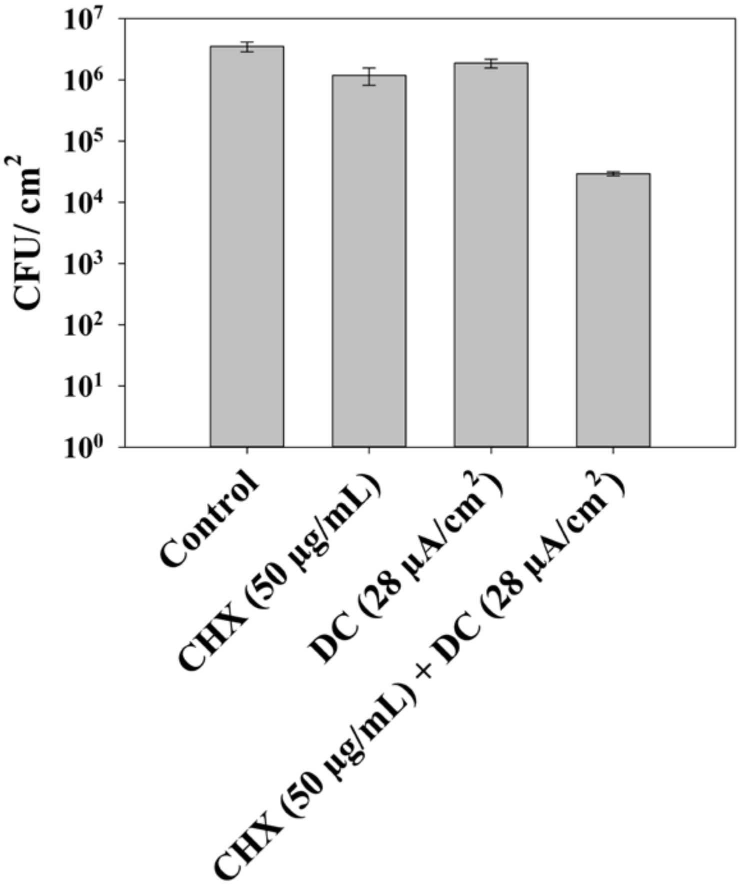

Figure S1. Viability of *S. aureus* biofilm cells after 1 h treatment with 50 µg/mL CHX alone, 28 µA/cm^2 DC alone or concurrent treatment with CHX and DC. The treatments were tested in a mixture of 0.85 % NaCl and artificial saliva medium (2:1, v/v).

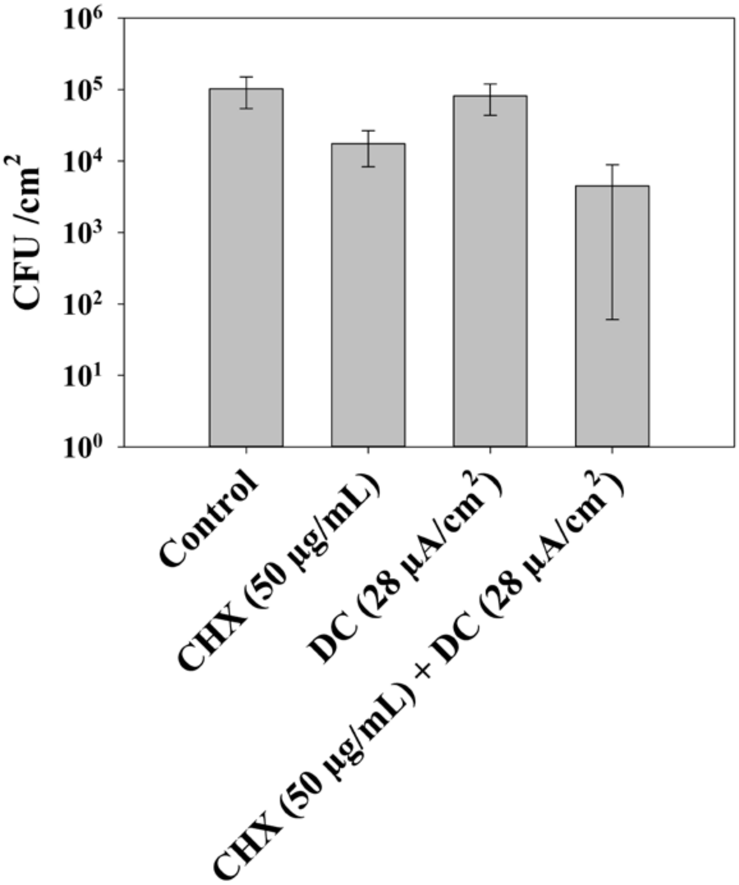

Figure S2. Viability of *S. mutans* biofilm cells after 1 h treatment with 50 µg/mL CHX alone, 28 µA/cm^2 DC alone or concurrent treatment with CHX and DC. The treatments were tested in a mixture of 0.85 % NaCl and artificial saliva medium (2:1, v/v).
